# Comparison of oscillometric, Doppler and invasive blood pressure measurement in anesthetized goats

**DOI:** 10.1371/journal.pone.0197332

**Published:** 2018-05-23

**Authors:** Olga Szaluś-Jordanow, Michał Czopowicz, Agata Moroz, Marcin Mickiewicz, Magdalena Garncarz, Emilia Bagnicka, Tadeusz Frymus, Jarosław Kaba

**Affiliations:** 1 Department of Small Animal Diseases with Clinic, Faculty of Veterinary Medicine, Warsaw University of Life Sciences, Warsaw, Poland; 2 Laboratory of Veterinary Epidemiology and Economics, Faculty of Veterinary Medicine, Warsaw University of Life Sciences, Warsaw, Poland; 3 Department of Pathology and Veterinary Diagnostics, Faculty of Veterinary Medicine, Warsaw University of Life Sciences, Warsaw, Poland; 4 Institute of Genetics and Animal Breeding, Polish Academy of Sciences, Jastrzębiec, Magdalenka, Poland; The University of Tokyo, JAPAN

## Abstract

Arterial blood pressure (BP) can be measured directly using an invasive intra-arterial method. This method is considered a gold standard, however it is potentially hazardous and requires expensive equipment and professional skills. Therefore, two non-invasive methods–Doppler ultrasonic sphygmomanometry and oscillometry–have been introduced in veterinary medicine. Their accuracy has so far been reliably evaluated in various animal species, however only one study included a small group of goats. Therefore, we carried out a large-scale study which aimed to evaluate agreement between the two non-invasive methods and invasive intra-arterial BP measurement in anesthetized goats at various age. The study included 122 goats of two Polish local breeds (Polish White Improved and Polish Fawn Improved): 67 adult females, 35 adult males, and 20 two-month-old female kids. Goats were anesthetized with the intravenous mixture of xylazine and ketamine. BP was measured simultaneously with the three methods in each goat with 7 measurements on average taken. The study showed that according to the criteria of the American College of Veterinary Internal Medicine (ACVIM) oscillometric method yielded BP measurements sufficiently consistent with invasive intra-arterial method in anesthetized adult goats– 95% of oscillometric BP measurements were expected to differ from invasive BP measurements by at most ±20–25 mmHg. The agreement was worse in goat kids–oscillometry significantly overestimated invasive BP measurements, which resulted in highly asymmetrical 95% limits of agreement. Doppler systolic BP very poorly conformed to invasive systolic BP both in adult goats and in kids and all the ACVIM criteria were violated. Concluding, oscillometry, but not Doppler ultrasonic sphygmomanometry, may be regarded as an alternative to invasive BP measurement in large-scale scientific studies involving adult goats, however, individual oscillometric BP measurements should be treated with caution as estimated 95% limits of agreement were wide.

## Introduction

Arterial blood pressure (BP) measurement on a peripheral artery provides a vital piece of medical information. Four BP measurements are used in human medicine: systolic (SBP), diastolic (DBP), mean (MBP) and pulse pressure (PP). Their routine use in veterinary is hindered by anxiety of animal patients, which precludes obtaining accurate and reliable records [1]. This handicap, however, does not apply to unconscious animals, and BP is commonly monitored during general anesthesia. Direct intra-arterial BP measurement is considered a reference method [2] even though it is known in humans to overestimate systolic aortal blood pressure by several mmHg [3]. However, it is an invasive procedure, which not only requires professional skills but also bears considerable risk of complications. Therefore, non-invasive methods have been gradually introduced in veterinary medicine, of which Doppler ultrasonic sphygmomanometry and oscillometry have been most extensively studied in various animal species. The American College of Veterinary Internal Medicine (ACVIM) has developed validation criteria for assessing the agreement between non-invasive and invasive BP measurements in veterinary medicine [4]: the mean difference (bias) of paired measurements for SBP and DBP treated separately is supposed to be ≤10 mmHg with a standard deviation (precision) of ≤15 mmHg, and 50% of SBP and DBP measurements should lie within 10 mmHg of the invasive method, and 80% within 20 mmHg of the invasive method in groups of at least 8 subjects. They are less strict than criteria applied to evaluation of non-invasive methods in human medicine developed by the US Association for the Advancement of Medical Instrumentation (AAMI) and the British Hypertension Society (BHS), which, however, compare automatic non-invasive methods with the mercury standard cuff/stethoscope auscultatory BP measurements.

Doppler ultrasonic method was the first non-invasive method widely applied in veterinary medicine, and technically is the closest to the cuff/stethoscope auscultatory method. Recently, it has been found consistent with invasive BP measurement in anesthetized rabbits [5], but previous results in horses [6, 7], pigs [8], dogs [9] and cats [10, 11] had been disappointing. Moreover, poor agreement between this technique and oscillometry has been observed in conscious dogs [12, 13], cats [14] and goats [15].

Oscillometry has proven to be acceptably consistent with invasive BP measurement in anesthetized dogs [9, 16], cats [10], horses [17], and also in 20 adult sheep [18]. On the other hand, Almeida et al. (2014) found oscillometry insufficiently accurate in the study on 13 anaesthetized adult sheep [19]. Also Aarnes et al. (2014) showed violation of ACVIM validation criteria in anaesthetized sheep, goats and cattle of various body weight, however the small ruminant group consisted of only 12 goats and 8 sheep and the two species were merged together for analytical purposes 20.

The goat has been used as an experimental in vivo model of various orthopedic conditions and surgical procedures in human medicine [21–24]. This results in growing need for safe general anesthesia in goats, for which the knowledge of the most appropriate techniques of BP monitoring is invaluable. Therefore, in view of paucity of literature regarding BP measurement in goats, we carried out a large-scale study which aimed to evaluate of agreement between two non-invasive methods (oscillometry and Doppler ultrasonic sphygmomanometry) and invasive intra-arterial BP measurement in anesthetized goats at various age.

## Materials and methods

### Animals

Data were collected from 122 goats of two Polish local breeds (Polish White Improved and Polish Fawn Improved): 67 adult females (55%; 66% of all adult goats) aged from 1 to 12 years with the median of 4 years and IQR from 3 to 7 years, 35 adult males (29%; 34% of all adult goats) aged 2 years (n = 19) or 3 years (n = 15), and 20 two-month-old female kids (16%). Body weight of adult goats ranged from 20 to 70 kg, and kids weighed between 7 and 10 kg.

Goats came from four dairy herds and all were intended for culling due to symptomatic caprine arthritis-encephalitis (adult goats) or chronic arthritis of unknown origin (kids). BP measurements were taken during general anesthesia directly preceding euthanasia. The study was approved by the II Local Commission for Experiments in Animals in Warsaw (WAW2/67/2016).

### BP measurement

Neither food nor water was withheld before anesthesia. A catheter was placed in the cephalic vein and mixture of xylazine (Xylapan, Vetoquinol, Poland) at dose of 0.05 mg/kg and ketamine (VetaKetam, VetAgro, Poland) at dose of 5 mg/kg in one syringe was given intravenously. No tracheal tube was inserted, only visual monitoring of respiration was used. An animal was placed in right lateral recumbency. A 20 gauge catheter was placed in the right femoral artery after shaving of hair, cleansing of the skin and making a 1 cm incision of the skin above the palpable pulse of the artery. The catheter was connected via saline-filled non-compliant tubing with a 3-way stopcock to a pressure transducer (ICU Medical, USA). The transducer was placed at the level of the right atrium of the heart and the system was electronically calibrated and zeroed to atmospheric pressure according to manufacturer’s recommendations (GE Dash 4000). The system was connected to a bag containing heparinized saline and was periodically flushed to maintain patency. The tubing was constantly checked for the presence of air bubble which were removed if present.

Oscillometric blood pressure was measured using PetTrust Veterinary blood pressure monitor for cat and dog (BioCare, Aster Electrical Co. Ltd., Taiwan). The cuff was placed in the middle of the left forearm and its width was fitted using the measure attached to the device. Doppler blood pressure was measured using Doppler Ultrasonic Flow Detector Model 811-B was used (Parks Medical Electronics, Inc.). Doppler blood pressure cuff was placed above the left hock. The limb circumference was measured and a cuff that was approximately 40% of the circumference of the limb was chosen [25]. The probe was placed above the dorsal metatarsal artery. Both invasive and non-invasive measurements were recorded simultaneously in a goat.

Invasive mean blood pressure was used to classify goats into blood pressure classes of normotensive (MBP for 65 to 100 mmHg), hypotensive (MBP <65mmHg), and hypertensive (MBP >100mmHg)[26]. Pulse pressure (PP) was calculated as a difference between SBP and DBP.

### Statistical analysis

Numerical variables were presented as the arithmetic mean and standard deviation (SD) or as the median and interquartile range (IQR), if SD exceeded one-half the arithmetic mean (so the variable was considered non-normally distributed). The range was presented in all cases. Comparisons between groups of unpaired observations were performed using the one-way analysis of variance (ANOVA) with the Tukey’s HSD post-hoc test for unequal group sizes, or Kruskal-Wallis H test with Dunn’s post-hoc procedure. Paired groups were compared using paired-sample Student’s t-test or Wilcoxon signed-rank test, the latter with a Bonferroni correction if more than 2 groups were analyzed. All statistical test were two-sided and the significance level (α) was set at 0.05.

Among measurements taken from each goat the outliers were identified according to Tukey’s rule with fences set at 3 interquartile ranges (IQR) above the upper quartile (Q3) or below the lower quartiles (Q1). Then variability of measurements obtained from a goat was calculated as the coefficient of variation (CV% = 100% × SD / arithmetic mean). Outliers were dropped from further analysis only if raised CV% above 20% [4]. Remaining measurements were averaged and their arithmetic mean served as the final systolic, diastolic and mean blood pressure value for each goat.

Agreement between blood pressure by invasive (I), oscillometric (O) and Doppler (D) method was determined by calculating the arithmetic mean of difference between pairs of methods (henceforth referred to as bias) ± SD (precision). Relationship between BPs and HR was determined using the Pearson’s product-moment correlation coefficient (r). Invasive method was regarded as a reference gold standard, and oscillometric and Doppler method as alternative tests. The 95% confidence interval (95% CI) for the bias was also calculated and if it fell entirely above or below 0 the results obtained using an alternative method were considered as significantly biased i.e. they were found to non-coincidently over- or underestimate the true blood pressure. To evaluate the clinical importance of the discrepancies the cumulative percentage of oscillometric and Doppler readings falling within ±10 mmHg and ±20 mmHg of the invasive reference method were determined. Furthermore, paired measurements were plotted on the scatter diagram (O vs. I and D vs. I) with the line of equality and then their differences (I-O, and I-D) were plotted against invasive BP measurements (not against the averages of the two methods as invasive method was considered a gold standard [27] to show the Bland-Altman limits of agreement (LoA), which were computed using the formula: LoA = bias ± 1.96 × precision, along with their 95% CIs [28]. LoAs create the prediction interval allowing to foresee by what maximum figures (in mmHg) the blood pressure values obtained using an alternative method may be expected to differ from true blood pressure in 95% of examinations.

The measures of agreement obtained were compared with ACVIM guidelines [4]. Power of these comparisons in which LoAs exceeded limits of ±30 mmHg accepted by ACVIM was calculated according to the method based on the non-central t-Student distribution [29]. Statistical analyzes were performed in Statistica 12 (StatSoft Inc., Tulsa, OK) and Microsoft Office Excel.

## Results

Using each of the three methods between 5 and 9 measurements (median of 7) were taken from each goat. In 35 adult goats (34%) and 3 kids (15%), despite repeated attempts lasting at least half an hour, we were unable to obtain Doppler blood pressure measurements since we could not localize the dorsal metatarsal artery with the Doppler probe. Median CV% ranged from 3.2% in invasive MBP to 8.8% in oscillometric DBP and in general variability of measurements was lower in invasive than in oscillometric and Doppler method, and was the highest in DBP (S1 File). Only one BP measurement was identified as an outlier and removed.

Kids had significantly lower SBP, DBP and MBP than adults when measured using invasive method, however this difference was not observed in oscillometry. Blood pressure did not differ between adult males and females regardless of the method used (Table 1).

**Table 1:** Systolic (SBP), diastolic (DBP) and mean (MBP) blood pressure and pulse pressure (PP) by invasive and oscillometric method in three animal groups–adult female goats (AF), adult male goats (AM) and female goat kids (K).

Blood pressure	Adult females (AF)	Adult males (AM)	Kids (K)	ANOVA p-value	Tukey’s post-hoc test p-value
	(n = 67)	(n = 35)	(n = 20)		
**Invasive method**					
SBP	111.9 ± 18.3	111.9 ± 20.1	96.2 ± 12.3	0.003*	AF vs. AM– 0.999
	(71–172)	(78–153)	(69–124)		AF vs. K– 0.019*
					AM vs. K– 0.019*
DBP	66.1 ± 11.9	64.2 ± 14.8	53.0 ± 8.8	<0.001*	AF vs. AM– 0.811
	(39–107)	(42–103)	(37–72)		AF vs. K– 0.003*
					AM vs. K– 0.013*
MBP	83.1 ± 14.0	81.1 ± 15.8	70.7 ± 10.4	0.003*	AF vs. AM– 0.830
	(54–131)	(61–118)	(51–90)		AF vs. K– 0.017*
					AM vs. K– 0.052*
PP	45.8 ± 11.5	47.7 ± 9.6	43.2 ± 5.8	0.099	-
	(24–88)	(31–71)	(32–51)		
**Oscillometric method**					
SBP	109.7 ± 19.7	115.8 ± 14.6	107.7 ± 18.1	0.184	-
	(63–164)	(89–152)	(82–149)		
DBP	62.3 ± 15.3	64.0 ± 16.2	62.8 ± 14.7	0.872	-
	(38–108)	(36–100)	(42–96)		
MBP	78.1 ± 16.1	81.0 ± 14.9	77.7 ± 14.4	0.633	-
	(46–128)	(53–117)	(56–113)		
PP	47.5 ± 9.9	51.8 ± 9.4	44.9 ± 14.6	0.047	-^b^
	(25–75)	(35–69)	(30–97)		
**Doppler method** ^a^					
SBP	93.6 ± 26.8	86.8 ± 21.0	74.5 ± 27.1	0.072	-
	(41–163)	(20–144)	(31–127)		

* significantly different at significance level of 0.05

^a^ measured in 34 AF, 33 AM and 17 K

^b^ all pairwise with the Tukey post-hoc test comparisons were insignificant at α = 0.05

Significant bias was observed between invasive and oscillometric blood pressure both in adults and in kids, however the direction and magnitude of the bias was different: in kids SBP, DBP and MBP were overestimated by oscillometry by 7–11 mmHg on average, whereas in adults oscillometry underestimated DBP and MBP significantly but only by 2–3 mmHg on average. In turn, oscillometric SBP and PP in adults were not significantly biased (Table 2). Oscillometry significantly underestimated DBP and MBP in normotensive goats, and MBP in hypertensive goats. The remaining six oscillometry BP measurements (all in hypotensive, SBP and DBP in hypertensive and SBP in normotensive) were not significantly biased (Table 3).

**Table 2:** Overall agreement between invasive and oscillometric systolic (SBP), diastolic (DBP), mean (MBP) blood pressure, and pulse pressure (PP) as well as between invasive and Doppler systolic (SBP) blood pressure in adult goats and in kids.

	Method			Absolute difference between invasive and oscillometric blood pressure [mmHg]	
Blood pressure [mmHg]	Invasive	Oscillometric	Mean difference (bias) ± SD of the difference (precision) (CI 95%) and p-value of the paired-sample Student’s t-test	≤10	≤20
Adult goats (n = 102)					
SBP	111.9 ± 18.8	111.8 ± 18.3	0.1^a^ ± 11.5^a^	68%^a^	93%^a^
	(71–172)	(63–164)	(-2.2, 2.3)		
			p = 0.939		
DBP	65.4 ± 12.9	62.8 ± 15.5	2.6^a^ ± 9.9^a^	72%^a^	98%^a^
	(39–107)	(36–108)	(0.7, 4.5)		
			p = 0.009*		
MBP	82.4 ± 14.6	79.0 ± 15.7	3.4 ± 9.3	78%	97%
	(54–131)	(46–128)	(1.5, 5.2)		
			p<0.001*		
PP	46.4 ± 10.9	49.0 ± 9.9	-2.5 ± 10.8		
	(24–88)	(31–71)	(-4.6, -0.4)		
			p = 0.021*		
SBP by Doppler (n = 67)	112.7 ± 20.8	90.3 ± 24.1	22.4^b^ ± 29.2^b^	18%^b^	28%^b^
	(71–172)	(20–163)	(15.3, 29.5)		
			p<0.001*		
Kids (n = 20)					
SBP	96.2 ± 12.3	107.7 ± 18.1	-11.5^b^ ± 11.1^a^	60%^a^	85%^a^
	(69–124)	(82–149)	(-16.7, -6.3)		
			p<0.001*		
DBP	53.0 ± 8.8	62.8 ± 14.7	-9.8^a^ ± 13.5^a^	50%^a^	80%^a^
	(37–72)	(42–96)	(-16.1, -3.5)		
			p = 0.004*		
MBP	70.7 ± 10.4	77.7 ± 14.4	-7.0^a^ ± 10.2^a^	70%	90%
	(51–90)	(56–113)	(-11.8, -2.3)		
			p = 0.006*		
PP	43.2 ± 5.8	44.9 ± 14.6	-1.7 ± 12.6		
	(25–75)	(35–69)	(-7.6, 4.2)		
			p = 0.564		
SBP by Doppler (n = 17)	95 ± 12.7	74.5 ± 27.1	21.0^b^ ± 23.5^b^	25%^b^	45%^b^
	(69–124)	(31–127)	(8.9, 33.1)		
			p = 0.002*		

^a^ the American College of Veterinary Internal Medicine (ACVIM) criterion satisfied

^b^ the American College of Veterinary Internal Medicine (ACVIM) criterion violated

* significant bias at the significance level of 0.05

**Table 3:** Agreement between invasive and oscillometric systolic (SBP), diastolic (DBP) and mean (MBP) blood pressure depending on the blood pressure class in adult goats.

Class of blood pressure according to invasive MBP	n	Mean difference (bias) ± SD (precision) and 95% confidence interval (in parentheses) for the bias between invasive and oscillometric blood pressure [mmHg]		
		SBP	DBP	MBP
Normotension (invasive MBP 65–100 mmHg)	79	0.6^a^ ± 10.3^a^	2.7^a^ ± 10.5^a^	3.5^a^ ± 9.6^a^
		(-1.8, 2.9)	(0.3, 5.1)	(1.3, 5.7)
		p = 0.630	p = 0.025*	p = 0.002*
Hypotension (invasive MBP <65 mmHg)	11	-7.0^a^ ± 11.9^a^	1.4^a^ ± 6.2^a^	-0.2^a^ ± 6.5^a^
		(-15.0, 1.0)	(-2.8, 5.6)	(-4.6, 4.2)
		p = 0.080	p = 0.468	p = 0.913
Hypertension (invasive MBP >100 mmHg)	12	3.5^a^ ± 16.3^b^	3.0^a^ ± 8.8^a^	5.8^a^ ± 9.0^a^
		(-6.9, 13.8)	(-2.6, 8.6)	(0.2, 11.5)
		p = 0.477	p = 0.257	p = 0.045*

^a^ the American College of Veterinary Internal Medicine (ACVIM) criterion satisfied

^b^ the American College of Veterinary Internal Medicine (ACVIM) criterion violated

* significant difference between invasive and oscillometric blood pressure in paired-sample Student’s t-test at significance level of 0.05

Oscillometry satisfied all the criteria of ACVIM in adults and in kids, except for the bias of SBP which was by 1.5 mmHg too high than accepted by ACVIM (Table 2) as well as in goats of all three pressure classes, except for the precision of SBP which was by 1.3 mmHg too high than accepted by ACVIM (Table 3).

LoAs indicated that 95% of oscillometric SBP measurements in adult goats were expected to be from 22 mmHg higher to 23 mmHg lower than invasive measurements, while in kids from 33 mmHg higher to only 10 mmHg lower than invasive measurements (S1 Fig). Similar results were obtained also for the remaining three BPs: oscillometric DBP measurement– 95% likely to be from 17 mmHg higher to 22 mmHg lower than invasive measurements in adults, while in kids from 36 mmHg higher to 17 mmHg lower than invasive measurements (S2 Fig); oscillometric MBP measurement– 95% likely to be from 15 mmHg higher to 22 mmHg than invasive measurements in adults, while in kids from 27 mmHg higher to 13 mmHg lower than invasive measurements (S3 Fig); and oscillometric PP measurement– 95% likely to be from 24 mmHg higher to 19 mmHg lower than invasive measurements in adults, while in kids from 26 mmHg higher to 23 mmHg lower than invasive measurements (S4 Fig). Detailed values of LoAs between invasive and oscillometric BP measurements with 95% CIs are presented in S2 File.

Doppler SBP poorly conformed to invasive SBP both in adult goats and in kids (Table 2). LoAs were very wide: from -34.7 (95% CI: -22.4, -47.1) to 79.6 (95% CI: 67.2, 91.9) mmHg in adults and from -25.1 (95% CI: -4.2, 46.1) to 67.1 (95% CI: 46.2, 88.1) mmHg in kids (S5 Fig). Power of the comparison was 98.2% in adult goats and 93.1% in kids. All criteria of ACVIM were violated.

Oscillometric and invasive HR was differed on average by -0.8±5.2 bpm and the bias was not significantly different from 0 (95% CI: 0.1, -1.8; p = 0.087). LoAs were from -11 (95% CI: -10, -13) to 10 (95% CI: 8, 11) bpm. No significant correlation was observed between any of BPs and HR (r from -0.13 to 0.13 for oscillometric BPs and from -0.46 to -0.10 for invasive BPs, detailed data not shown).

## Discussion

We showed in our study that oscillometric method yielded BP measurements sufficiently consistent with invasive intra-arterial method in anesthetized adult goats according to ACVIM criteria. The ACVIM criteria were satisfied also in each of blood pressure classes except for SBP in hypertension, whose SD was, however, only slightly above 15 mmHg (16.3 mmHg). Ninety five per cent of oscillometric BP measurements are expected to differ from invasive BP measurements by at most 20–25 mmHg, which is less than allowed by ACVIM guidelines (1.96 × SD of 15 mmHg which is ±30 mmHg from the bias between invasive and non-invasive method; [4]). Agreement in our study was better than showed in adult sheep [18, 19] or in the mixed group of adult goats and sheep [20] between invasive BP on the auricular arteria and oscillometry on the left thoracic limb. Nevertheless, the identical tendencies were observed: in the three aforementioned studies, like in ours, SBP was not significantly biased (the mean difference of 0), while both DBP and MBP were significantly underestimated by oscillometry. Still, biases we observed were much lower than observed by the others (DBP: 2 mmHg vs. 10–16 mmHg; MBP: 3 mmHg vs. 7–10 mmHg), which resulted in virtually symmetrical LoAs, while the LoAs obtained by the others were considerably skewed.

The agreement was worse in goat kids, in which presented LoAs must be treated with even higher caution as the sample of kids was much smaller than adult goats. Oscillometry significantly overestimated invasive BP measurements (contrary to the bias observed in DBP and MBP in adults), which resulted in highly asymmetrical LoAs. This may indicate that the cuff used was undersized. As we used a tape measure provided by the manufacturer of the oscillometric device to determine the cuff size to be used, it appears that perhaps in goat kids wider cuff than recommended should be chosen by default.

It ought to be stressed that the maximum difference between 95% of BP measurements of ±30 mmHg is twofold more than AAMI allows in terms of automatic devices used in human medicine (1.96 × SD of 8 mmHg is 16 mmHg) [30]. Given that normal SBP, DBP and MBP in goats are 90–130 mmHg, 60–90 mmHg, and 70–110 mmHg, respectively [31], and hypotension in anesthetized goat is defined as MBP lower than 65 mmHg [26], so wide range of expected differences raises concerns regarding clinical applicability of oscillometric BP measurement in adult goats. In our opinion oscillometry is an useful method as the measurement may be obtained easily and immediately, however BP measurements close to limits indicating life-threatening conditions should be interpreted cautiously and preferably verified using the invasive method. Moreover, we think that oscillometry is not a reasonable alternative to the invasive method in scientific studies quantifying relationships between BP and other variables or studying BP-independent effects of any intervention or variable in which goats act as a model for human medicine, especially when small groups are enrolled. This opinion is in line with previous conclusions made in the case of laboratory animals [32]. On the other hand, application of oscillometry can be considered in large-scale studies which focus on group- rather than individual-level effects. Given that systematic biases we observed were low (<5 mmHg), and precision was also quite good (roughly 10 mmHg), oscillometry seems to provide quite good overall estimate of BP for a group of goats.

Our study undoubtedly disqualifies Doppler ultrasonic sphygmomanometry as an accurate method of SBP measurement in anesthetized goats. It is not because we could not localize pulse on the dorsal metacarpal artery and measure SBP in a considerable proportion of goats as switching to another vessel might have perhaps solved this problem. However, LoAs were significantly beyond the ACVIM acceptable range and Doppler tended to overestimate SBP considerably both in adults and kids. Large number of adult goats enrolled in the study makes this conclusion highly trustworthy, however the discrepancies observed were so big that even the sample size of 17 kids resulted in the probability of error below 10% (power of analysis of 93%). Along with our previous results showing large disparities between oscillometric and Doppler BP measurements in conscious goat [15], this observation ultimately shows that Doppler ultrasonic method must not be recommended as an alternative to invasive BP measurement in goats.

This is the first study to evaluate BP measurements in a group of males which was large enough to fulfil AAMI requirements (at least 30% of all individuals included), and to compare BP between sexes. BP proved not to differ between males and females, contrary to previous observations in dogs [33], but in agreement with observations in cats [34]. This is also the first study to compare BP between young and adult small ruminants. Kids had lower BP than adults, which is in line with what is considered normal in people [35], and previous observations in growing beagle dogs [36]. However, this could only be noted using an invasive method, not oscillometry. This observation indicates that problems with selection of the best cuff size may considerably distort the truth, what has already been shown in children [37] and laboratory animals [32].

In our study a different artery (femoral) was used for invasive BP measurement compared to previous studies in small ruminants (auricular). The femoral artery is more central artery than auricular, so it could perform slightly better as an indicator of aortal BP, as MBP measurements from the auricular artery have been shown to be lower than those obtained from the carotid artery in rabbits [38]. Nevertheless, we believe that any variations resulting from this difference may be dismissed as unimportant, and these results can be considered as comparable.

Invasive method proved to yield significantly more repeatable BP measurements (as indicated by significantly lower CV%) than oscillometry and Doppler. Generally, the range of CV% observed was wide although quite similar to previously obtained [19] and DBP was recorded with greater variability than the other two BPs (S1 File). On the other hand, both the CV% and the number of outliers identified in this study were lower than in the study carried out before in conscious goats [15], which implies that alleviation of goat anxiety by anesthesia improves the repeatability of BP measurements.

Concluding, oscillometry, but not Doppler ultrasonic sphygmomanometry, may be regarded as an alternative to invasive BP measurement in large-scale scientific studies involving adult goats, however, individual oscillometric BP measurements should be treated with caution.

## Supporting information

S1 Fig Agreement between invasive and oscillometric systolic blood pressure (SBP) in adult goats (○; n = 102) and kids (+; n = 20). Diagonal line on the scatter plot (A) is a line of equality. Upper and lower solid horizontal lines on Bland-Altman plots (B) signify the upper and lower limits of agreement, respectively while the broken lines show the mean difference (i.e. bias) between invasive and oscillometric SBP measurements.
(TIF)

S2 Fig Agreement between invasive and oscillometric diastolic blood pressure (DBP) in adult goats (○; n = 102) and kids (+; n = 20). Diagonal line on the scatter plot (A) is a line of equality. Upper and lower solid horizontal lines on Bland-Altman plots (B) signify the upper and lower limits of agreement, respectively, while the broken lines show the mean difference (i.e. bias) between invasive and oscillometric DBP measurements.
(TIF)

S3 Fig Agreement between invasive and oscillometric mean blood pressure (MBP) in adult goats (○; n = 102) and kids (+; n = 20). Diagonal line on the scatter plot (A) is a line of equality. Upper and lower solid horizontal lines on Bland-Altman plots (B) signify the upper and lower limits of agreement, respectively while the broken lines show the mean difference (i.e. bias) between invasive and oscillometric MBP measurements.
(TIF)

S4 Fig Agreement between invasive and oscillometric pulse pressure (PP) in adult goats (○; n = 102) and kids (+; n = 20). Diagonal line on the scatter plot (A) is a line of equality. Upper and lower solid horizontal lines on Bland-Altman plots (B) signify the upper and lower limits of agreement, respectively while the broken lines show the mean difference (i.e. bias) between invasive and Doppler PP measurements.
(TIF)

S5 Fig Agreement between invasive and Doppler systolic blood pressure (SBP) in adult goats (○; n = 67) and kids (+; n = 17). Diagonal line on the scatter plot (A) is a line of equality. Upper and lower solid horizontal lines on Bland-Altman plots (B) signify the upper and lower limits of agreement, respectively while the broken lines show the mean difference (i.e. bias) between invasive and Doppler SBP measurements.
(TIF)

S1 File Variability of blood pressure measurements (systolic, diastolic and mean) obtained using three different methods (I-invasive, O-oscillometric and D-Doppler) in 122 goats given as the coefficient of variability (CV%)–median, IQR and range. (DOCX)

S2 File Limits of agreement (LoA) with 95% confidence interval (95% CI) in brackets between invasive and oscillometric method determined separately for 102 adult goats and 20 kids. (DOCX)
