# Supplementary figures and images for: Comparison of oscillometric, Doppler and invasive blood pressure measurement in anesthetized goats
Source: PLoS One. 2018 May 23;13(5):e0197332. doi: 10.1371/journal.pone.0197332 (PMC5965870; doi:10.1371/journal.pone.0197332)

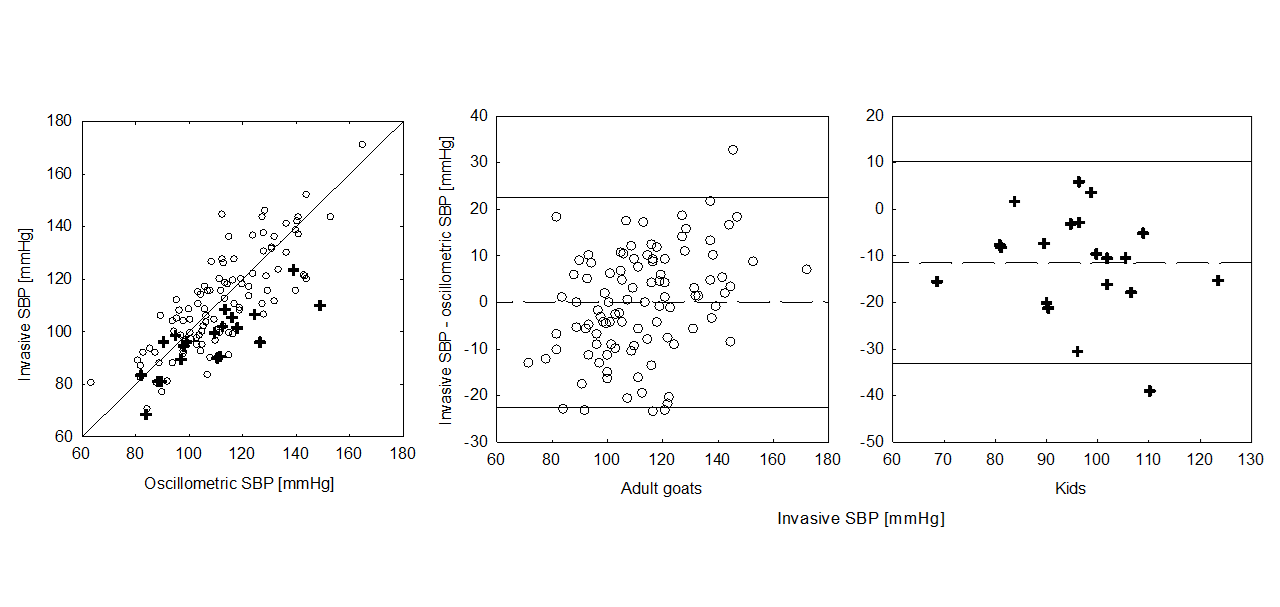

Supplement: S1 Fig — Diagonal line on the scatter plot (A) is a line of equality. Upper and lower solid horizontal lines on Bland-Altman plots (B) signify the upper and lower limits of agreement, respectively while the broken lines show the mean difference (i.e. bias) between invasive and oscillometric SBP measurements. (TIF) [file pone.0197332.s001.tif]

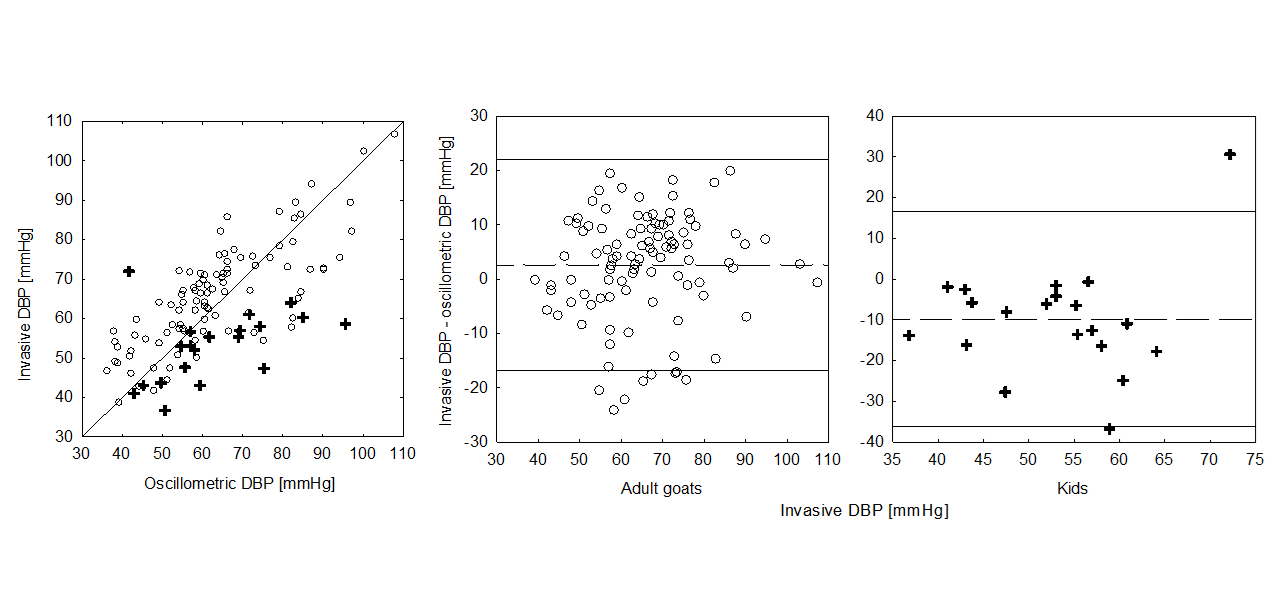

Supplement: S2 Fig — Diagonal line on the scatter plot (A) is a line of equality. Upper and lower solid horizontal lines on Bland-Altman plots (B) signify the upper and lower limits of agreement, respectively, while the broken lines show the mean difference (i.e. bias) between invasive and oscillometric DBP measurements. (TIF) [file pone.0197332.s002.tif]

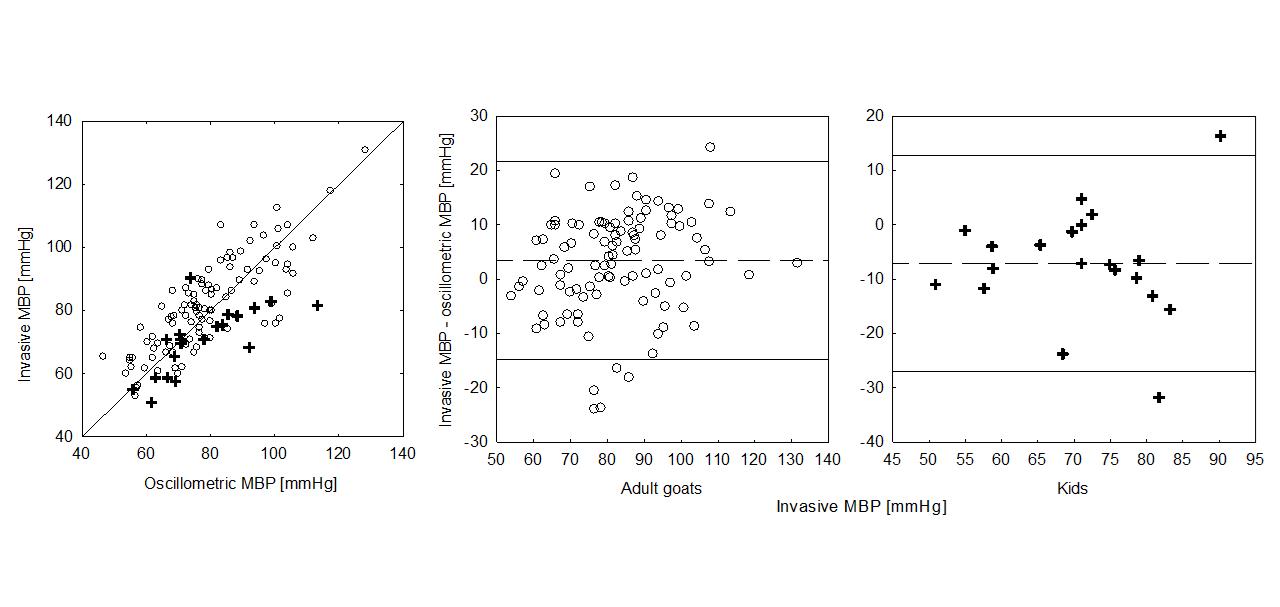

Supplement: S3 Fig — Diagonal line on the scatter plot (A) is a line of equality. Upper and lower solid horizontal lines on Bland-Altman plots (B) signify the upper and lower limits of agreement, respectively while the broken lines show the mean difference (i.e. bias) between invasive and oscillometric MBP measurements. (TIF) [file pone.0197332.s003.tif]

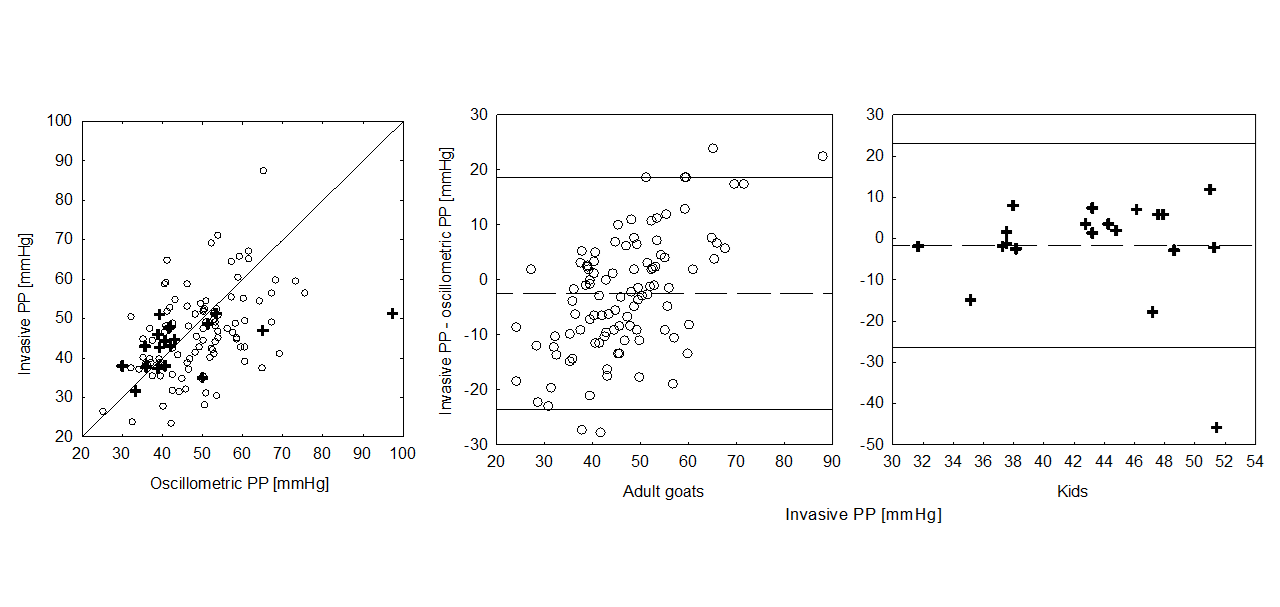

Supplement: S4 Fig — Diagonal line on the scatter plot (A) is a line of equality. Upper and lower solid horizontal lines on Bland-Altman plots (B) signify the upper and lower limits of agreement, respectively while the broken lines show the mean difference (i.e. bias) between invasive and Doppler PP measurements. (TIF) [file pone.0197332.s004.tif]

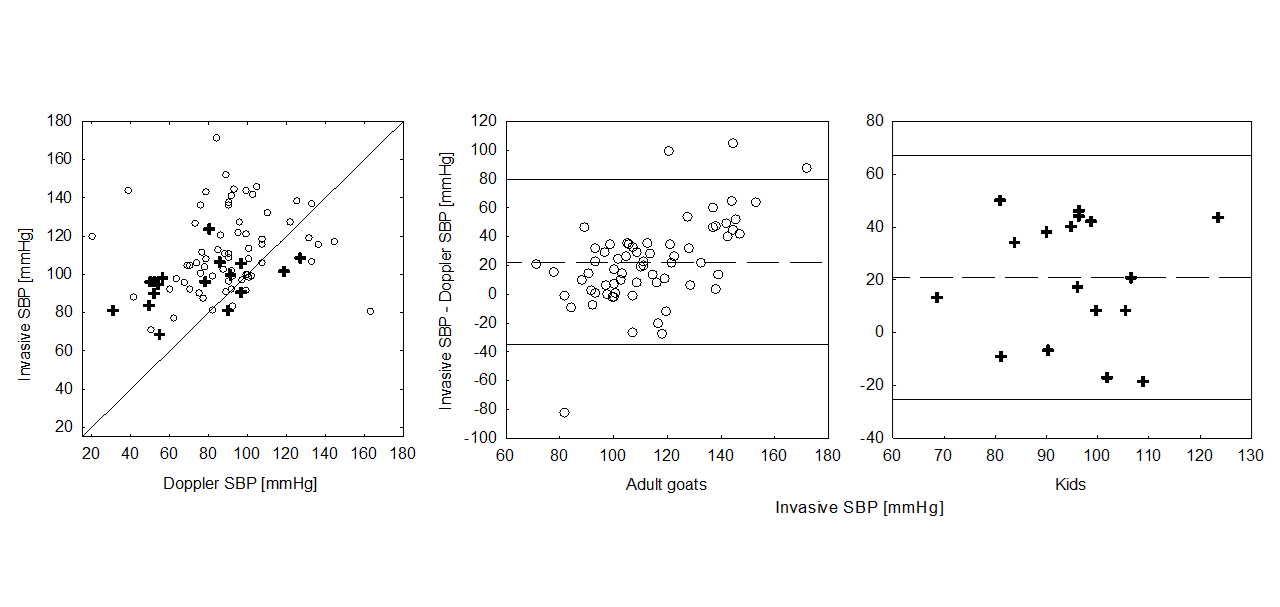

Supplement: S5 Fig — Diagonal line on the scatter plot (A) is a line of equality. Upper and lower solid horizontal lines on Bland-Altman plots (B) signify the upper and lower limits of agreement, respectively while the broken lines show the mean difference (i.e. bias) between invasive and Doppler SBP measurements. (TIF) [file pone.0197332.s005.tif]
