# Supplementary material for: Comparison of oscillometric, Doppler and invasive blood pressure measurement in anesthetized goats
Source: PLoS One. 2018 May 23;13(5):e0197332. doi: 10.1371/journal.pone.0197332 (PMC5965870; doi:10.1371/journal.pone.0197332)
Supplement: S1 File — (DOCX) [file pone.0197332.s006.docx]

Supplementary file 1. Variability of blood pressure measurements (systolic, diastolic and mean) obtained using three different methods (I-invasive, O-oscillometric and D-Doppler) in 122 goats given as the coefficient of variability (CV%) – median, IQR and range

| Blood pressure | Invasive (I) | Oscillometric (O) | Doppler (D) | Wilcoxon signed rank test  p-value |
| --- | --- | --- | --- | --- |
| Systolic (SBP) | 3.7%, 2.1%-6.3%  (0%-35.0%) | 5.9%, 3.9%-8.9%  (0.8%-27.0%) | 6.2%, 3.9%-8.5%  (0%-17.9%) | I vs. O <0.001^a*^  I vs. D 0.021^a*^  O vs. D 0.294^a^ |
| Diastolic (DBP) | 4.4%, 2.4%-7.2%  (0%-45.5%) | 8.8%, 5.8%-12.6%  (2.5%-45.5%) | - | <0.001 |
| Mean (MBP) | 3.3%, 1.7%-5.8%  (0.5%-34.9%) | 6.2%, 3.9%-9.8%  (1.7%-34.5%) | - | <0.001 |
| Kruskal-Wallis test and Dunn’s test  p-value | 0.012  SBP vs. DBP 0.398  SBP vs. MBP 0.417  DBP vs. MBP 0.009^*^ | <0.001  SBP vs. DBP <0.001^*^  SBP vs. MBP 0.999  DBP vs. MBP <0.001^*^ | - | - |

^a^ with Bonferroni correction (i.e. p-value × 3)

* significant at α=0.05
