# Supplementary material for: Comparison of oscillometric, Doppler and invasive blood pressure measurement in anesthetized goats
Source: PLoS One. 2018 May 23;13(5):e0197332. doi: 10.1371/journal.pone.0197332 (PMC5965870; doi:10.1371/journal.pone.0197332)
Supplement: S2 File — (DOCX) [file pone.0197332.s007.docx]

Supplementary file 2. Limits of agreement (LoA) with 95% confidence interval (95% CI) in brackets between invasive and oscillometric method determined separately for 102 adult goats and 20 kids

| Blood pressure | LoA (95% CI) between invasive and oscillometric method in mmHg | | | |
| --- | --- | --- | --- | --- |
|  | Adult goats | | Kids | |
|  | Lower LoA | Upper LoA | Lower LoA | Upper LoA |
| SBP | -22.4 (-18.5, -26.3) | 22.6 (18.7, 26.5) | -33.1 (-42.1, -24.2) | 10.2 (1.2, 19.1) |
| DBP | -16.8 (-13.4, -20.2) | 22.0 (18.6, 25.3) | -36.2 (-25.3, -47.2) | 16.6 (5.7, 27.5) |
| MBP | -14.8 (-11.7, -18.0) | 21.6 (18.4, 24.7) | -26.9 (-18.7, -35.2) | 12.9 (4.6, 21.1) |
| PP | -23.6 (-20.0, -27.3) | 18.6 (14.9, 22.3) | -26.4 (16.2, -36.6) | 23.1 (12.8, 33.3) |
